# Disaster preparedness behaviour of tourist village managers in Mount Merapi, Indonesia

**DOI:** 10.4102/jamba.v17i1.1914

**Published:** 2025-08-15

**Authors:** Oktomi Wijaya, Indri H. Susilowati, Neil Towers

**Affiliations:** 1 Faculty of Public Health, Universitas Indonesia, Depok, Indonesia; 2 Department of Occupational Health and Safety, Faculty of Public Health, Universitas Ahmad Dahlan, Yogyakarta, Indonesia; 3 Department of Occupational Health and Safety, Faculty of Public Health, Universitas Indonesia, Depok, Indonesia; 4 Gloucestershire Business School, University of Gloucestershire, Gloucestershire, United Kingdom

**Keywords:** predisposing, enabling, reinforcing, preparedness, tourist village managers, Mount Merapi

## Abstract

Disaster preparedness behaviour in Indonesia is still considered low. The purpose of this study is to understand the factors that influence the disaster preparedness behaviour of tourist village managers around Mount Merapi. This study is a qualitative study with a phenomenological approach. Data were collected through a focus group discussion (FGD), with 32 participants in 3 sessions with 10–11 participants per session. The data were analysed using Collaizi’s approach by reading the transcripts to obtain an overview and insight, extracting important statements, formulating meaning using codes and grouping into themes. We examined factors influencing disaster preparedness of tourist village managers in Mount Merapi using the PRECEDE component guide from the PRECEDE-PROCEED model. The results of this study indicated that seven themes were found related to the disaster preparedness factors of tourist village managers: four themes related to predisposing factors, namely, belief, knowledge, risk perception and experience; two themes related to enabling factors, namely, availability of infrastructure and training; and one theme related to reinforcing factors, namely, support from various parties.

**Contribution:**

By knowing all three factors that drive behaviour – predisposing, enabling and reinforcing factors – interventions can be more comprehensive, targeting not only the initial motivation for behaviour change but also the necessary resources and ongoing support for sustainability. This holistic approach is critical to achieving behavioural change in disaster preparedness behaviour among tourism village managers.

## Introduction

Mount Merapi is the most active and dangerous volcano in the world (Jousset, Pallister & Surono 2013; Surono et al. 2012). Mount Merapi has erupted 61 times since the 15th century with a return period of every 3.5 years (Thouret et al. 2000) and causes severe disruption and danger to life. Mount Merapi is located 25 kilometres north of the city of Yogyakarta and is inhabited by around 1.6 million people. The volcano lies above the Java subduction zone (Surono et al. 2012). Eruptions during the 20th century usually recur every 4–6 years. The most devastating eruption of Mount Merapi in this century occurred in 2010, displacing at least a third of a million people and claiming nearly 400 lives (Jousset et al. 2013; Maly, Iuchi & Nareswari 2015). The eruption of Mount Merapi has had a severe impact on the agriculture, dairy farming and tourism sectors (Garcia-Fry et al. 2022).

The eruption of Mount Merapi has significantly affected the livelihood adaptation pattern of the community. The southern region of Mount Merapi, such as the subdistricts of Cangkringan, Pakem and Turi, which was severely affected by the 2010 eruption, experienced a change in livelihoods from livestock to tourism services because of the increased health risks and changes to the ecosystem and economy (Umaya et al. 2020). Almost all people in rural areas around Merapi diversified their sources of income, assets and activities (Sagala, Okada & Paton 2009). This is one of the ways to reduce risk and respond to disasters. The decision to diversify into non-agricultural occupations such as tourism can be one of the risk mitigation and coping strategies for frequent disasters (Barrett, Reardon & Webb 2003).

One of the many tourist destinations currently developing in the area around Mount Merapi is the creation of tourist villages. A tourist village is not a new concept, with the idea originating in France, a country that has a long tradition of organising and promoting various forms of rural tourism (Ciolac n.d.). A tourist village is an area that has the potential and uniqueness of a typical tourist attraction, namely experiencing the unique life and traditions of rural communities with all its potential with the principle of active community involvement in the management of tourism activities (Wirdayanti et al. 2021).

Currently, around the Mount Merapi area in Sleman Regency Yogyakarta, there are 32 tourist villages in different stages of development. The development of the tourist village concept is part of tourism development initiatives in the area of Mount Merapi, which has beautiful natural panoramas, cool weather, high biodiversity, as well as supporting water and land resources, creating a green and healthy environment (Pramono & Ashari 2015). Tourism in the Merapi Volcano disaster-prone area is not completely prohibited but rather restricted and monitored by the Centre for Research and Development of Geological Disaster Technology and the Local Disaster Management Agency. Tourism activities are regulated to prevent entry into extremely dangerous zones (within a 3 km radius of the summit). Tourism is the primary source of income for communities around Merapi, especially following major eruptions like the one in 2010. A complete ban on tourism activities could have a significant impact on the local economy, which is heavily reliant on tourists.

The development of tourism villages in the Mount Merapi region has both positive and negative impacts. The development of tourism villages has boosted the local economy. A study (Hastuti, Purwantara & Khotimah 2008) found that tourist villages can contribute to poverty alleviation by utilising local resources and involving local communities. A study (Zaroh 2022) showed that the establishment of tourism villages has led to an increase in employment, income and the quality of facilities and infrastructure in the community. On the contrary, the development of tourist villages around Mount Merapi can also increase disaster risk because of the position of these tourist villages in eruption-prone areas.

In an effort to reduce disaster risk in the Mount Merapi area, it is necessary to improve community preparedness. The results of several studies on community preparedness in the area around Merapi show that the level of community preparedness is still at the unprepared level. A study (Wijaya, Galih & Putri 2023) showed that the preparedness of Mount Merapi tourist village managers is still low. Tourist village managers do not yet have contingency plans and evacuation procedures for tourists in the event of an eruption. The study by Rivani and Mei (2022) also showed the same results: the community’s preparedness is still low, especially in terms of guidelines, emergency response plans, resource mobilisation and disaster management infrastructure. Disaster management planning in tourist villages is crucial to ensure the safety of tourists. Disaster management in tourist villages is supported by the local community with leadership from the tourist village manager. Based on the results of a pilot study, it was found that some tourist village managers around Mount Merapi do not have a disaster management plan in place when an eruption of Mount Merapi occurs. For instance, tourist village managers do not have procedures for evacuating tourists in the event of an eruption.

**FIGURE 1:**
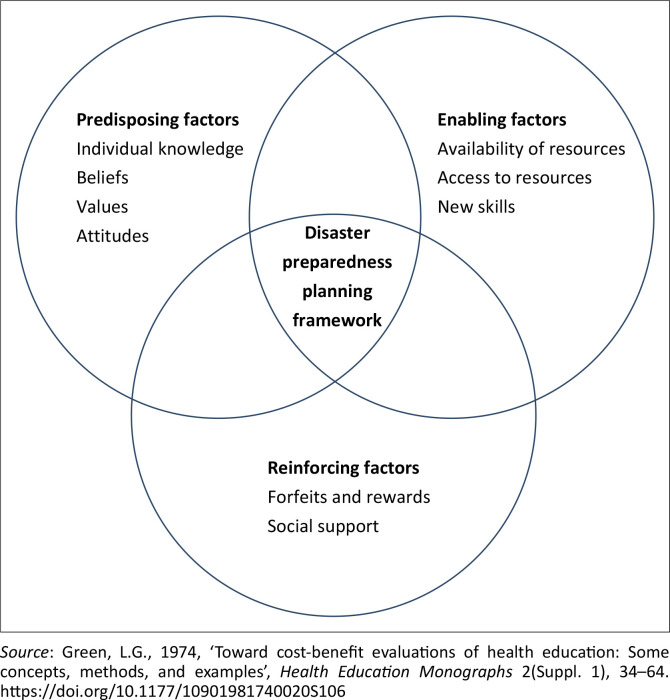
Disaster preparedness planning conceptual framework.

In the absence of a disaster mitigation plan, it is important to understand what factors influence disaster preparedness behaviour among tourist village managers around Mount Merapi. According to the PRECEDE-PROCEED Model, a person’s behaviour is influenced by three factors: predisposing, enabling and reinforcing (PRECEDE) (Kegler & Miner 2004). Predisposing factors are individual characteristics that motivate a person to behave, including individual knowledge, beliefs, values and attitudes. Enabling factors are factors or characteristics of the environment that facilitate action, including the availability and access to resources and new skills needed for behaviour change. Reinforcing factors are factors that reinforce or encourage behaviour. Reinforcing factors include punishments or rewards given as a consequence of behaviour. Reinforcing factors also include social support (Green 1999). The factors influencing disaster preparedness among tourism village managers in the disaster-prone area of Mount Merapi are shown in Figure 1. The purpose of this study is to understand the PRECEDE factors that influence the disaster preparedness planning process for tourist villages in the event of an eruption of Mount Merapi.

## Research methods and design

This study used a phenomenological approach with data collected through the focus group discussion (FGD) method.

### Study area

This study was conducted in the disaster-prone area of Mount Merapi, located on the border between the province of Central Java and the Special Region of Yogyakarta, Indonesia. It is the most active volcano in Indonesia and has erupted regularly since 1548. There are currently 32 tourist villages on the slopes of this volcano, which is one of the most popular tourist attractions in the Yogyakarta region. It has a beautiful natural panorama, specific climatic characteristics and high biodiversity, and there are supporting tourism resources, especially water and land resources, and a fertile and healthy environment.

### Sampling and recruitment

One respondent was selected from each tourist village community, such as the tourist manager, community leadership committee member or other members of the community with experience of actual eruptions. The following selection criteria were used: experience in disaster planning for at least 1 year, willingness to attend at a predetermined time (FGDs were scheduled at three different times, 20–22 November 2023) and willingness to participate in the FGDs. Each FGD session was recommended to consist of 6–12 participants (Seal, Bogart & Ehrhardt 1998). Evidence showed that three FGD groups were sufficient to identify relevant themes in the data set (Guest, Namey & McKenna 2017). A total of 32 participants engaged in this study, which was conducted in three sessions with 10–11 participants per session.

### Study procedures

Before the FGDs began, each participant was asked for their willingness to participate in this study by filling out an informed consent form. The FGDs were recorded using an audio recorder and they were moderated by one research member using FGD guidelines. Participants were given material on the PRECEDE model regarding factors that influence human behaviour. According to Green and Kreuter (2005), there are three factors that influence behaviour, namely, PRECEDE factors. The questions asked in this FGD were:

- In your opinion, what are the characteristics of individuals that can influence the preparedness of tourism village managers in dealing with Mount Merapi eruptions?
- In your opinion, what resources are needed by tourism village managers to improve their preparedness for Mount Merapi eruptions?
- In your opinion, what external factors can strengthen the preparedness of tourism village managers in dealing with Mount Merapi eruptions?

The FGD was conducted in three sessions, and each FGD session lasted 60–90 min.

### Data analysis

Audio recordings of the FGDs were transcribed. Then the transcripts of the FGD results were reviewed and analysed. The data were analysed using Collaizi’s approach (Praveena & Sasikumar 2021). There were four steps in analysing phenomenological data. The first step was to obtain an overview of the transcripts by reading the transcripts many times so that they can be clearly understood. In a study on the preparedness of tourism village managers in the disaster-prone area of Mount Merapi, researchers conducted direct interviews, which helped to gain a comprehensive understanding of the participants’ overall opinions. The audio recordings were listened to three times in an effort to understand the participants’ thought processes, opinions and views. The second step was to extract important statements from the transcripts that form the overall meaning of the experience. Researchers extracted important phrases and statements from the transcripts that together formed the overall meaning of the opinion or viewpoint. I reread the transcripts and analysed each one to identify important statements. These statements were written separately for each participant and coded with the transcript page number and line number. This was given to members of the peer group for review in order to obtain clarity of thought, and the suggestions were taken into consideration. The third step was to formulate the meaning of these important statements. At this stage, researchers attempt to formulate more general statements or meanings for each important statement in the text. These meanings are formulated from important statements and discussed with members of the same peer group. After obtaining the formulated meaning of the important statements, the researcher grouped these meanings into theme clusters. These theme clusters were then reduced to emerging themes. The PRECEDE constructs in the PRECEDE-PROCEED model were used to analyse the themes.

### Ethical considerations

Ethical clearance to conduct this study was obtained from the Research Ethics Committee Universitas Ahmad Dahlan (KEP UAD), with ethical approval number: 012307107.

## Results

The discussion with informants asked about the following:

- In your opinion, what are the individual factors that can influence the preparedness of tourism village managers in dealing with the eruption of Mount Merapi?
- In your opinion, what resources are needed by tourism village managers to deal with the eruption of Mount Merapi?
- In your opinion, what external factors can strengthen the preparedness of tourism village managers in dealing with the eruption of Mount Merapi?

A total of seven themes were identified from the FGD transcripts. We categorised the themes based on the three PRECEDE factors, the components in the PRECEDE-PROCEED framework. Seven themes were found related to the preparedness factors of tourist village managers: four themes related to predisposing factors, namely, belief, knowledge, risk perception and experience; two themes related to enabling factors, namely, availability of infrastructure and training; and one theme related to reinforcing factors, namely, support from various parties.

### Predisposing factors

There are four themes associated with predisposing factors: belief, knowledge, risk perception and experience.

Tourist village managers around Mount Merapi have the perception that community belief is a factor that influences disaster preparedness. Tourist village managers believe that Mount Merapi has two opposing considerations. On the one hand, Mount Merapi is a threat that can cause a severe disaster if an eruption occurs. On the other hand, Mount Merapi is considered a blessing and livelihood because the eruption of Merapi can cause the land to become fertile. Thus, they have the belief to coexist with nature:

‘We just live side by side with Mount Merapi, when there is an eruption we have to evacuate, when conditions are safe we still live near Mount Merapi, because our source of livelihood is there.’ (Tourism Village Manager 3)

Furthermore, the tourist village managers assume that knowledge is an important aspect that affects disaster preparedness in the community. Communities around Mount Merapi have local knowledge passed down from generation to generation about the signs of Mount Merapi about to erupt. The signs that Mount Merapi is about to erupt are usually marked by the descent of animals such as monkeys, deer and tigers into community settlements. These signs will alert managers that Mount Merapi might erupt, and they need to increase preparedness:

‘It is common knowledge that if animals such as monkeys, deer and tigers have descended on villages, this is a bad sign that Mount Merapi will erupt.’ (Tourism Village Manager 7)

Risk perception is also believed by tourist village managers to be a factor that affects disaster preparedness. There are differences in perceptions between managers whose tourist villages are close to the peak of Mount Merapi and managers whose tourist villages are quite far from Mount Merapi. Managers whose tourist village is close to the peak of Mount Merapi have the perception that their tourist village is in a dangerous area:

‘Turgo is the closest tourist village to the peak of Mount Merapi. We live in a dangerous area. Thus, we realise that we must always improve our preparedness and always monitor information on the status of Mount Merapi because we live in a disaster-prone area.’ (Tourism Village Manager 22)

Meanwhile, managers whose tourist villages are quite far from the peak of Mount Merapi have the perception that the location of their tourist villages is safe from the dangers of Mount Merapi:

‘The location of our tourist village is quite far from the peak of Mount Merapi, so residents feel safe and not worried about the threat of eruption.’ (Tourism Village Manager 11)

Previous disaster experience is also considered by tourist village managers as a factor influencing disaster preparedness. Tourist village managers who have had much experience in dealing with eruptions have understood the patterns and characteristics of the eruption of Mount Merapi:

‘We have experienced the eruption of Merapi from 1994, we also understand the characteristics and patterns of the eruption of Mount Merapi, besides that we also know the dangerous and safe areas.’ (Tourism Village Manager 32)

### Enabling factors

There are two themes related to enabling factors, namely infrastructure and training. Tourist village managers argue that the availability of disaster management infrastructure is a factor that can improve preparedness in the event of an eruption of Mount Merapi. Tourist village managers highlight the importance of the availability of evacuation route infrastructure and communication tools:

‘The availability of infrastructure is important to improve preparedness, especially communication tools to update the latest information on the status of Mount Merapi.’ (Tourism Village Manager 1)

‘The availability of infrastructure such as evacuation routes, evacuation signs, and adequate means of transport will facilitate the evacuation process if an eruption occurs.’ (Tourism Village Manager 18)

In addition to the availability of infrastructure, tourist managers believe that training is a factor that can influence community preparedness. Tourist managers argue that communication and information training is very important and needed to improve preparedness in facing the threat of Mount Merapi eruption:

‘The most urgent thing is communication and information training in disaster management, how to convey information to the public if there is an increase in the status of Mount Merapi.’ (Tourism Village Manager 14)

### Reinforcing factors

There is one theme related to reinforcing factors, namely, support and cooperation with various stakeholders. Tourist managers argue that preparedness for the threat of a Mount Merapi eruption requires coordination and collaboration with various parties:

‘Improving Mount Merapi’s eruption preparedness requires support from various parties such as the village government, Disaster Preparedness Cadets, the Centre for Geological Disaster Technology Research and Development [*BPPTKG*], the Regional Disaster Management Agency, and the Tourism Office.’ (Tourism Village Manager 29)

## Discussion

Although there have been many studies that discuss disaster preparedness in communities and the factors that influence it, there are still limited studies that discuss disaster preparedness factors using the PRECEDE-PROCEED Model. We examined the factors that influence the disaster preparedness of tourist village managers in Mount Merapi. Using the PRECEDE component guide in the PRECEDE-PROCEED model, seven themes on disaster preparedness factors were found: four themes related to predisposing factors, namely, belief, knowledge, risk perception and experience; two themes related to enabling factors, namely, availability of infrastructure and training; and one theme related to reinforcing factors, namely, support from various parties.

Beliefs play an important role in driving preparedness behaviour. Moreover, people identified as having strong beliefs in the effectiveness of disaster preparedness have preparedness behaviours at 7% to 30% higher levels compared to those with weaker preparedness beliefs (Thomas et al. 2015). In the context of Mount Merapi, tourist village managers believe that Mount Merapi has two contrasting dimensions: it is both a blessing and a potential disaster. Tourist village managers believe that they live in a disaster-prone area. Beliefs related to the inevitability of threats, the effectiveness of preparedness and self-efficacy influence the adoption of preparedness behaviours (Najafi et al. 2017).

Good disaster knowledge has a positive and significant correlation with disaster preparedness (Setyawati et al. 2020; Wulandari et al. 2023). Communities around Mount Merapi have local knowledge about the signs that Mount Merapi will erupt, namely the descent of animals such as monkeys, deer and tigers into residential areas. This local knowledge can be used by tourist village managers in the vigilance against the eruption of Mount Merapi.

Related to risk perception, tourist village managers closer to the summit of Mount Merapi have different perceptions. Managers whose tourist villages are located near the peak of Mount Merapi realise that they live in a dangerous area, while managers whose tourist villages are located quite far from the peak of Mount Merapi have the perception that the place they live is still safe from the threat of Mount Merapi eruption. Risk perception plays an important role in influencing preparedness behaviour. Research consistently shows that when risk perception increases, individuals are more likely to adopt behaviours that are perceived to reduce that risk. This is because when people perceive a risk as unacceptable, they tend to engage in behaviors they believe are most effective in minimizing that risk and achieving the best possible outcome (Goddard et al. 2018). In the context of Mount Merapi, more attention is needed for tourist villages that are quite far from the peak of Mount Merapi to ensure that they do not neglect vigilance measures in their planning process because they think they live in a safe area.

Previous experiences with disasters or emergencies have been found to correlate with the behaviour of residents during disasters, including the implementation of preparedness measures. Tourist village managers in Mount Merapi who have had previous experience of eruption disasters have understood the various patterns and characteristics of Mount Merapi eruptions. Some evidence suggests that previous emergency or disaster experiences can motivate people to adopt desirable behavioural strategies, such as evacuating. Individuals who have previous disaster experience are better prepared compared to those who do not have previous disaster experience (Oral et al. 2015). This suggests that past experiences with disasters can significantly influence a person’s level of preparedness to deal with future disaster events.

Tourist village managers around Merapi assume that the availability of infrastructure and equipment can assist disaster preparedness. This is supported by research (Adini et al. 2009) that the level of infrastructure and equipment readiness has been shown to have a significant impact on the performance of emergency response teams during a disaster. Tourist village managers highlighted the importance of the availability of evacuation routes, means of transport and means of communication. The function of evacuation routes is to provide a safe and efficient route for people to leave an area in the event of a disaster. (Putri & Maryono 2018). In the context of the Mount Merapi region, evacuation routes are essential because of the risk of volcanic eruptions. These routes are designed to guide the population away from the volcano and towards designated shelters. (Hardiansyah et al. 2020). In addition, tourist village managers also highlighted the importance of the availability of effective communication, including radios appropriate for disaster management scenarios. A study (Hafida, Setiawan & Rose Anna 2018) showed that community radio in the Mount Merapi area was very effective in supporting disaster preparedness, with an effectiveness rate of 63.6%.

The results of the discussion showed that tourist village managers thought that training could influence disaster preparedness. Tourist village managers particularly highlighted the importance of providing risk communication training. Effective risk communication plays an important role in reminding individuals about the nature of hazards, the level of danger and the actions needed to protect themselves. Research has shown that improved risk communication tends to be positively correlated with preventive behaviour (Gammoh, Dawson & Katsikopoulos 2023).

Merapi tourism managers argued that disaster preparedness is strongly influenced by support from other partners associated with disaster preparedness and planning. Partners that need to be involved in improving the preparedness of tourism village managers are the village government, disaster preparedness cadres, the tourism office and the regional disaster management agency. Collaboration between organisations and government agencies is essential for the development of effective strategies and better performance during disasters (Pratama et al. 2020). Collaboration allows for the sharing of resources, knowledge and expertise, which can result in more comprehensive and effective disaster preparedness plans.

According to the PRECEDE-PROCEDE model, by identifying the factors that influence the preparedness behaviour of tourist village managers on Mount Merapi, intervention programme strategies can be formulated to increase the capacity of community preparedness.

## Conclusion and limitations

In this paper, we identify PRECEDE factors that affect the disaster preparedness of tourist village managers around Mount Merapi. Our work found that individual characteristic factors such as knowledge, risk perception, experience, availability of access to infrastructure, training and support from various parties are considered as factors that influence disaster preparedness. In accordance with the PRECEDE-PROCEED model, with the identification of these factors, a programme strategy to improve disaster preparedness of tourist village managers in Mount Merapi can be determined.

The limitation of the study is that this research employed a qualitative approach which relates to disaster preparedness in 32 tourist villages located in the Mount Merapi disaster-prone area in Indonesia. However, future studies can analyse the broader region and other types of disasters.
